# Fate of External Carotid Artery Following Carotid Artery Stenting for Internal Carotid Artery near Occlusion

**DOI:** 10.3390/biomedicines13020303

**Published:** 2025-01-26

**Authors:** Dorota Łyko-Morawska, Michał Serafin, Julia Szostek, Magdalena Mąka, Iga Kania, Wacław Kuczmik

**Affiliations:** Department of General Surgery, Vascular Surgery, Angiology and Phlebology, Faculty of Medical Sciences in Katowice, Medical University of Silesia, 45-47 Ziołowa Street, 40-635 Katowice, Poland; s81394@365.sum.edu.pl (J.S.); s81151@365.sum.edu.pl (M.M.); s80989@365.sum.edu.pl (I.K.); wkuczmik@interia.pl (W.K.)

**Keywords:** carotid artery stenting, internal carotid artery, external carotid artery, carotid artery near-occlusion

## Abstract

Background: The external carotid artery (ECA) plays a vital role in facial perfusion and acts as a collateral pathway for cerebral blood flow during internal carotid artery (ICA) stenosis. In cases of carotid near-occlusion (CNO), characterized by severe ICA stenosis with hemodynamic changes, carotid artery stenting (CAS) is rising as a potential new treatment. During CAS, the stent is deployed in ICA, covering the ECA orifice. Therefore, this study aims to evaluate the effects of CAS on ECA. Materials and Methods: This retrospective study included 159 patients diagnosed with CNO and treated with CAS between February 2018 and May 2023. Preoperative and postoperative ECA diameters were measured using angiography. Data on patient demographics, procedural details, and outcomes were analyzed. Results: The median preoperative ECA diameter was 4.34 mm, decreasing to 3.40 mm post-CAS (*p* < 0.001). ECA narrowing occurred in 76.39% of patients, while 4.17% experienced occlusion. A larger preoperative ECA diameter was predictive of narrowing (odds ratio (OR) = 1.35, *p* = 0.02) and protective against occlusion (OR = 0.1, *p* < 0.001). Weak correlations between ICA and ECA diameter changes were observed, indicating procedural influences on ECA dynamics. Conclusions: CAS for CNO significantly reduces ECA diameter, with a subset of patients developing occlusion. The preoperative ECA diameter is a key predictor of postoperative changes. These findings emphasize the need for further research on CAS-related ECA hemodynamic alterations to optimize patient outcomes and minimize complications.

## 1. Introduction

The external carotid artery (ECA), with its numerous branches, serves as the primary blood supplier to the facial region of the skull, providing critical perfusion to structures such as the face, scalp, and oral cavity [1,2]. Beyond its well-established role in facial blood supply, emerging evidence highlights the ECA’s significance as a collateral source for cerebral perfusion, particularly in pathological states involving the internal carotid artery (ICA). Several authors have underscored the ECA’s compensatory role in supplying the brain during ICA stenosis or occlusion, where it contributes to maintaining hemodynamic stability within cerebral circulation [3]. Studies estimate that up to 15% of the blood flow in the middle cerebral artery (MCA) may originate from the ECA under such conditions, reflecting its adaptability and importance in cerebrovascular compensation [4,5]. Despite these findings, the ECA remains frequently underappreciated in clinical assessments and interventional planning.

For many years, carotid endarterectomy (CEA) has been considered the gold standard for treatment of ICA stenosis. This procedure involves the removal of atherosclerotic plaque from the ICA and, in most cases, includes additional atherectomy of the ECA plaque [6]. As a result, CEA restores blood flow through both the ICA and ECA. With advancements in endovascular surgery, a less invasive alternative known as carotid artery stenting (CAS) was introduced, quickly gaining popularity as a viable treatment option. Clinical studies have demonstrated that CAS and CEA achieve comparable outcomes; therefore, the selection of a treatment method should be customized to the patient’s specific condition while also considering the surgeon’s expertise and preferences [6,7]. However, a notable distinction between CEA and CAS is the fate of ECA. During CAS, the stent is positioned within the ICA and extends into the bulb of the common carotid artery, frequently covering the orifice of the ECA, a phenomenon referred to as overstenting [3].

Previous studies conducted by the authors [8], as well as by other researchers [3,9,10], have demonstrated that overstenting can lead to abnormal blood flow in the ECA. This reduction in ECA blood flow has been associated with various clinical symptoms, including jaw claudication, craniofacial pain, and acute hemifacial ischemia [8,11,12]. Furthermore, ECA stenosis has been identified as a significant predictive factor for all-cause mortality, increasing the risk of death by approximately 2.6 times [13].

Recently, there has been a growing interest among vascular surgeons in the management of carotid artery near occlusion (CNO). According to the European Society for Vascular Surgery (ESVS) guidelines, CNO is defined as severe ICA stenosis accompanied by hemodynamic changes. Differentiation between CNO and severe ICA stenosis requires the observation of at least two out of four hemodynamic criteria: (1) delayed contrast filling distal to the stenotic ICA, (2) the presence of collateral blood supply to the brain, (3) a distal ICA diameter equal to or smaller than the diameter of the ipsilateral ECA, or (4) a distal ipsilateral ICA diameter smaller than that of the contralateral ICA [14]. Recent meta-analyses suggest that invasive treatment of CNO, including CAS, may offer superior outcomes compared to best medical treatment, resulting in improved clinical outcomes for patients [15,16]. Given the critical role of the ECA in compensatory mechanisms, especially in cases of severe ICA stenosis [4,5,8], and the limited understanding of the effects of CAS on the ECA in CNO, further investigation is needed.

Therefore, the primary aim of this study is to evaluate the impact of CAS on the ECA in cases of CNO. Additionally, this study seeks to identify the critical predictive factors that determine the outcome of the ECA following endovascular treatment of CNO. By examining these factors, this research aims to provide a better understanding of how CAS affects ECA hemodynamics and to identify potential risks or complications associated with overstenting, ultimately contributing to more personalized and effective treatment strategies for patients with CNO.

## 2. Materials and Methods

### 2.1. Study Characteristics

This retrospective study analyzed all patients admitted to the Department of General Surgery, Vascular Surgery, Angiology, and Phlebology at the Medical University of Silesia in Katowice, Poland, with a diagnosis of carotid artery stenosis between February 2018 and May 2023.

The inclusion criteria were patients with CNO confirmed by preoperative angiography who underwent primary treatment with CAS. Exclusion criteria included patients with carotid artery stenosis without near-occlusion, unsuccessful CAS (defined as failure to achieve stent deployment due to the technical or anatomical problems), and those treated for in-stent restenosis. These criteria ensured a focused study population by excluding secondary interventions and unsuccessful procedures, allowing for the evaluation of primary CAS influence on the ECA in CNO. 

The study group consisted of 159 patients (109 males and 50 females) with the median age of 69 years.

### 2.2. Equipment

Carotid artery angiography was conducted using the Artis zee ceiling system (Siemens Healthineers AG, Forchheim, Germany). During CAS procedures, both the 1st (The PRECISE PRO RX^®^ Carotid Stent System, Cordis, Miami Lakes, FL, USA) and 2nd (The CGuardTM Embolic Prevention System, InspireMD, Miami, FL, USA) generation carotid stents were utilized. The selection of stent type, along with its length and width, was determined by the lead vascular surgeon based on the specific requirements of each case.

### 2.3. CNO Diagnosis

**Figure 1:**
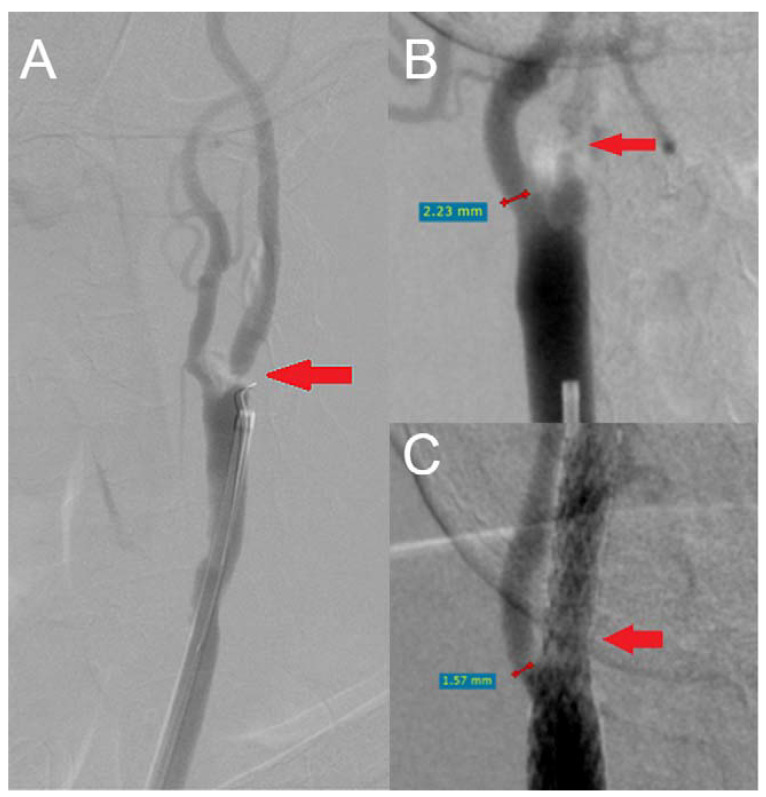
Carotid artery digital subtraction angiography with the measurement of external carotid artery (ECA) diameter. (**A**) Preprocedural angiograms. Red arrow: carotid artery near occlusion without distal vessel collapse. (**B**) Red line: preprocedural ECA measurement, Red arrow: internal carotid artery near occlusion with distal vessel collapse. (**C**) Red line: postprocedural ECA measurement, Red arrow: internal carotid artery with inserted stent (RadiAnt DICOM VIEWER, Medixant, Poznań, Poland).

CNO was diagnosed in preoperative angiography following the 2023 ESVS guidelines on carotid artery stenosis [14] using the RadiAnt DICOM VIEWER (Medixant, Poznań, Poland) version 2024.1 (Figure 1A).

### 2.4. Analyzed Data

Data on patient characteristics were obtained from the department’s electronic medical record (EMR) system. The following variables were analyzed: demographic information, comorbidities, and clinical symptoms.

Demographic data: Age and gender were recorded for each patient. Age was categorized as a continuous variable, and gender was categorized as male or female. Comorbidities: The presence of common comorbidities was assessed based on documented medical history and diagnosis codes within the EMR system. Comorbidities were classified as follows:

Arterial hypertension: Defined as a history of diagnosed hypertension or current use of antihypertensive medications.

Dyslipidemia: Defined as a documented history of abnormal lipid levels (cholesterol, triglycerides) or the current use of lipid-lowering medications (statins, etc.).

Diabetes mellitus: Identified as a diagnosis of type 1 or type 2 diabetes or the current use of antidiabetic medications (insulin, metformin, etc.). The presence of each comorbidity was recorded as a binary variable (yes/no).

Generalized atherosclerosis: Defined as a documented history of systemic atherosclerosis affecting multiple arteries, confirmed through imaging, clinical diagnosis, or both.

Coronary artery disease: Defined as a history of clinically diagnosed coronary artery disease, including evidence of coronary artery narrowing or previous revascularization procedures such as coronary artery bypass grafting (CABG) or percutaneous coronary intervention (PCI).

History of myocardial infarction: Identified as a documented history of myocardial infarction (heart attack), confirmed through clinical records, diagnostic tests (e.g., ECG, cardiac biomarkers), or both.

Clinical symptoms: Clinical symptoms were based on patient-reported symptoms documented in the EMR. Symptoms were categorized into several key clinical manifestations, including the following:

Stroke: Documented history of ischemic or hemorrhagic stroke.
Dizziness: Recorded as a symptom indicating vertigo, lightheadedness, or balance disturbances.
Headache: Recorded instances of primary or secondary headache types (e.g., migraine, tension, and cluster).
Transient Ischemic Attack (TIA): History of brief neurological symptoms consistent with a TIA.
Syncope: Documented history of fainting episodes or unexplained loss of consciousness.
Tinnitus: Self-reported ringing or buzzing in the ears.

Each clinical symptom was recorded as a binary variable (presence or absence). A composite variable was created to indicate the presence of any clinical symptoms, with a patient categorized as “symptomatic” if they had one or more of the listed symptoms.

Details of the CAS procedure, such as the type, width, and length of the stent used, were sourced from the procedural documentation.

Measurements of the ECA diameter were taken from preoperative and postoperative angiograms, specifically at the narrowest segment located between the ECA orifice and the origin of the superior thyroid artery (Figure 1B,C).

### 2.5. Statistical Analysis

Statistical analysis was conducted using Statistica^®^ software (version 13.3, StatSoft, Tulsa, OK, USA, 2013). Qualitative variables are presented as absolute values and percentages, while quantitative variables are expressed as ranges, means, standard deviations, or medians with interquartile ranges. The Shapiro–Wilk test was applied to assess the statistical distribution of the data. The Chi-square test was used to compare categorical variables between independent groups when the expected frequencies were sufficient (typically > 5 in each cell). When the expected frequencies were low, Fisher’s exact test was employed to examine the association between categorical variables in small sample sizes or when one or more expected frequencies were <5. Comparisons between independent groups for continuous variables that were not normally distributed were made using the Mann–Whitney U test. For dependent groups, the Wilcoxon signed-rank test was applied to compare related samples when the data did not follow a normal distribution. Spearman’s rank correlation coefficient was used to examine the strength and direction of the monotonic relationship between two continuous or ordinal variables when the assumptions of normality were not met. Predictive factors for ECA narrowing and occlusion were analyzed through univariate logistic regression. Confidence intervals (95%) were calculated for the odds ratios. A *p*-value of <0.05 was considered statistically significant.

## 3. Results

### 3.1. Descriptive Data of the Patients

The median age of the patients was 69 years. Our cohort was 109 (68.55%) males. Fifty-seven (35.85%) patients declared current cigarette smoking. Most (151; 94.96%) patients had comorbidities, with the most common being arterial hypertension (142; 89.31%). Clinical symptoms were reported by 108 (67.92%) patients, with a history of stroke being the most common (66; 41.55%) (Table 1).

### 3.2. Perioperative Data

**Figure 2:**
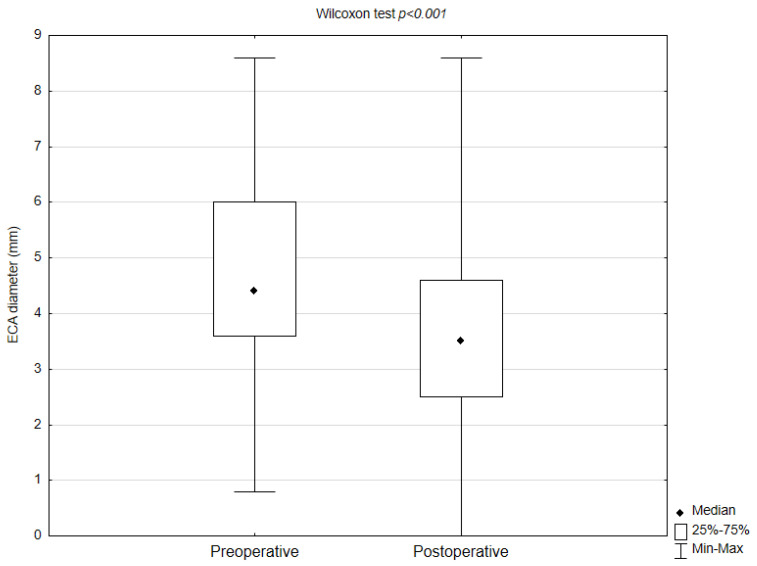
External carotid artery diameter before and after carotid artery stenting (Statistica 13.1, StatSoft, Tulsa, OK, USA).

The median ICA diameter measured 0.8 mm (IQR: 0.36 mm) preoperatively and increased to 5.60 mm (IQR: 2 mm) postoperatively. Most (80; 50.31%) of the procedures were performed in left ICA. The most (91; 57.23%) common stent used was the second-generation stent. Most (97; 61.01%) stents had 40 mm length, and the most common (64; 40.25%) stent width was 8 mm. Perioperative ECA occlusion was observed in seven (4.40%) patients. In most patients (144; 94.73%) the stent covered the orifice of the ECA. In 110 (76.39%) patients, the ECA diameter decreased after the procedure. The median ECA diameter measured 4.34 mm (IQR: 2.25 mm) preoperatively and decreased to 3.40 mm (IQR: 2 mm) postoperatively (Table 2). No significant differences in ECA diameter changes were observed between stent width, length, and generations (*p* = 0.39, *p* = 0.30, *p* = 0.13, respectively). There was a statistical significant difference between pre- and postoperative ECA diameter (*p* < 0.001) (Figure 2).

### 3.3. Correlations

**Figure 3:**
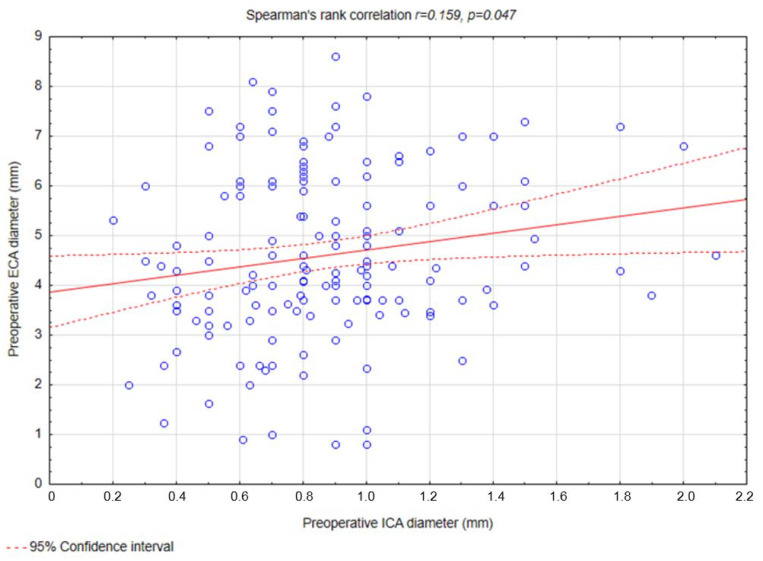
Spearman’s rank correlation coefficient between preoperative ICA diameter and ECA diameter. Abbreviations: ICA—internal carotid artery, ECA—external carotid artery.

**Figure 4:**
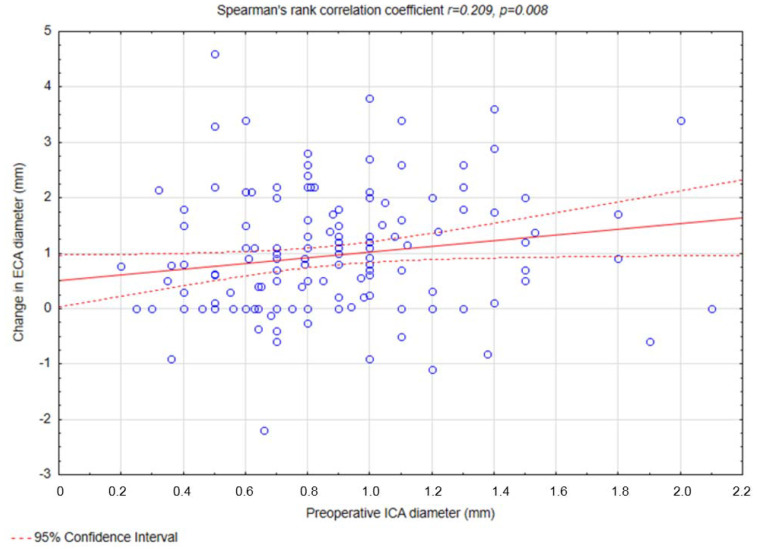
Spearman’s rank correlation coefficient between preoperative ICA diameter and change in ECA diameter. Abbreviations: ICA—internal carotid artery, ECA—external carotid artery.

**Figure 5:**
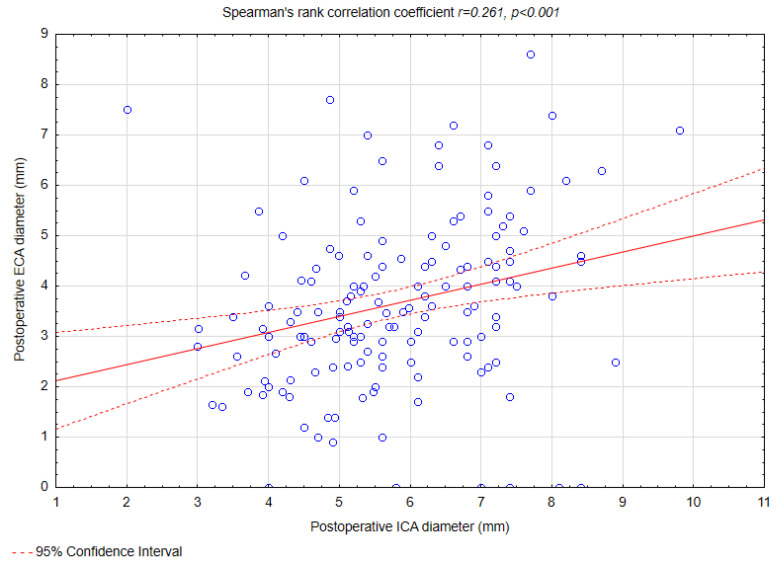
Spearman’s rank correlation coefficient between postoperative ICA diameter and change in ECA diameter. Abbreviations: ICA—internal carotid artery, ECA—external carotid artery.

**Figure 6:**
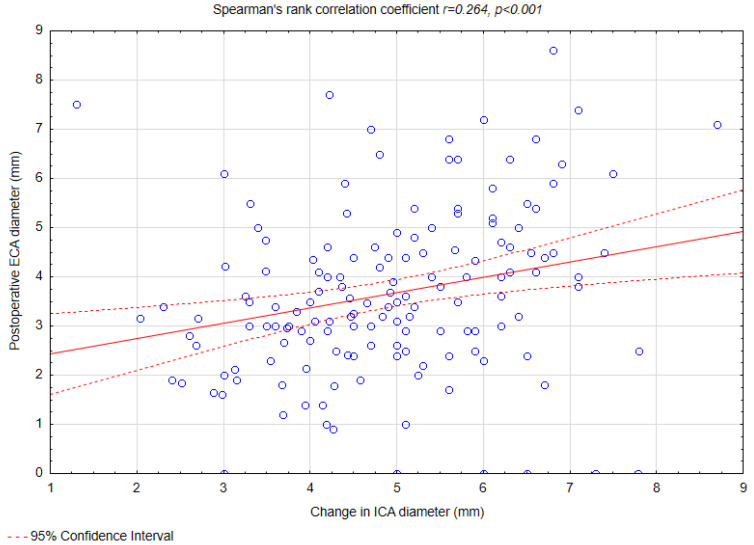
Spearman’s rank correlation coefficient between changes in ICA diameter and postoperative ECA diameter. Abbreviations: ICA—internal carotid artery, ECA—external carotid artery.

Weak correlations were observed between preoperative ICA diameter and ECA diameter (r = 0.159, *p* = 0.047) (Figure 3), preoperative ICA diameter and changes in ECA diameter (r = 0.209, *p* = 0.008) (Figure 4), postoperative ICA diameter and changes in ECA diameter (r = 0.261, *p* < 0.001) (Figure 5), and changes in ICA diameter and postoperative ECA diameter (r = 0.264, *p* < 0.001) (Figure 6).

No statistical significant correlations were found between postoperative ICA diameter and changes in ECA diameter (r= −0.024, *p* = 0.76) and between changes in ICA diameter and changes in ECA diameter (r = −0.06, *p* = 0.42).

### 3.4. Predictive Factors for Postoperative ECA Narrowing

**Figure 7:**
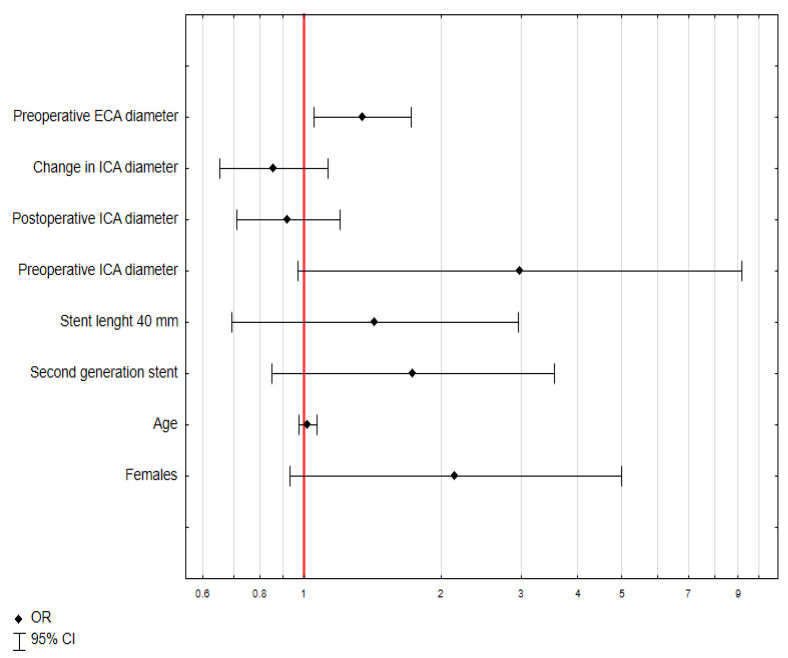
Univariate logistic regression analysis of predictive factors for postoperative ECA narrowing. Abbreviations: OR—odds ratio, 95% CI—95% confidence interval.

In univariate logistic regression analysis, only the greater ECA diameter is a positive predictive factor for postoperative ECA occlusion (odds ratio = 1.35, 95% confidence interval = 1.05–1.72, *p* = 0.02) (Figure 7).

### 3.5. Predictive Factors for Postoperative ECA Occlusion

**Figure 8:**
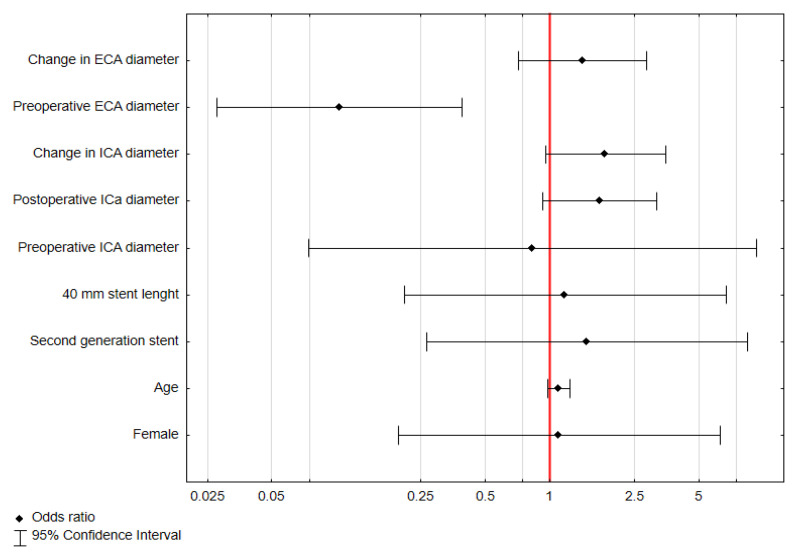
Univariate logistic regression analysis of predictive factors for postoperative ECA occlusion. Abbreviations: OR—odds ratio, 95% CI—95% confidence interval.

In univariate logistic regression analysis, only the greater ECA diameter are a negative predictive factor for postoperative ECA occlusion (odds ratio = 0.1, 95% confidence interval = 0.028–0.389, *p* < 0.001) (Figure 8).

## 4. Discussion

In this study, the external carotid artery (ECA) was significantly affected by carotid artery stenting (CAS). Preoperative ECA diameter had a median value of 4.34 mm, which decreased to 3.40 mm postoperatively, with 76.39% of patients showing a reduction in diameter. Interestingly, 4.17% of patients experienced ECA occlusion after the procedure. Statistical analysis revealed that the ECA diameter was significantly different between pre- and postoperative measurements (*p* < 0.001). A larger preoperative ECA diameter was identified as a significant predictive factor for postoperative ECA narrowing, with an odds ratio of 1.35 (*p* = 0.02), while greater ECA diameter where a negative predictive factor for postoperative ECA occlusion with OR of 0.1 (*p* < 0.01).

A reduction in ECA diameter was observed in 76.39% of patients in this study. This reduction was statistically significant (*p* < 0.001), with a median preoperative ECA diameter of 4.34 mm (IQR 2.25 mm) and a postoperative diameter of 3.40 mm (IQR 2 mm). To date, only the previous study by the authors [8], has specifically reported changes in ECA diameter following CAS. However, other studies have investigated alterations in ECA blood flow velocity after CAS. For example, Reichman et al. [17] reported an increase in peak systolic velocity (PSV) in the ECA from 161.7 cm/s preoperatively to 175.9 cm/s at 30 days and 216.7 cm/s at two years post-CAS. Furthermore, PSV in the ECA at all follow-up points was significantly higher than that observed following CEA. Additionally, an increased prevalence of ≥50% and ≥70% ECA narrowing post-CAS was noted, rising from 29.9% and 19.29% at three months to 65.8% and 38.32% at 60 and 24 months, respectively [3,18]. Due to the procedural characteristics of CAS, atherosclerotic plaque is often displaced toward the ECA orifice during balloon predilatation and/or postdilatation, originating from the CCA and ICA. Furthermore, the stent-induced disturbances in blood flow dynamics may contribute to a reduction in ECA diameter. These findings underscore the importance of further investigating ECA hemodynamics following CAS to better understand its implications and optimize patient outcomes.

Our study identified that a greater preoperative ECA diameter was predictive of a reduction in ECA diameter following CAS (OR = 1.35, *p* = 0.02). This finding aligns with the hypothesis that a wider ECA lumen may allow for a more pronounced displacement of atherosclerotic material during the procedure, thereby increasing the risk of narrowing or occlusion. Additionally, a weak positive correlation was observed between the change in ECA diameter and the postoperative ICA diameter (R = 0.261, *p* < 0.001), suggesting that procedural factors influencing ICA dilation, such as ballooning and stent placement, may simultaneously affect ECA geometry and blood flow dynamics. Moreover, we noted additional weak correlations that further elucidate the relationship between ICA and ECA changes. Specifically, weak positive correlations were found between preoperative ICA diameter and preoperative ECA diameter (R = 0.159, *p* = 0.047) and between preoperative ICA diameter and change in ECA diameter (R = 0.209, *p* = 0.008). Similarly, the change in ICA diameter was weakly correlated with postoperative ECA diameter (R = 0.264, *p* < 0.001). To the best of our knowledge, no study to this date has shown similar findings.

Nonetheless, the relationship between ICA stenosis and ECA hemodynamics has been explored by various researchers. Shmelev et al. [19] investigated potential correlations between ECA and ICA parameters, demonstrating that ECA peak systolic velocity (PSV) was moderately elevated in patients with ≥50% ICA stenosis compared to those with <50% stenosis. In cases with <50% ICA stenosis, a PSV threshold of 148 cm/s was effective in identifying ≥50% ECA stenosis. For patients with ≥50% ICA stenosis, a higher PSV cutoff of 179 cm/s provided the best accuracy in diagnosing ≥50% ECA stenosis. Additionally, Zakko et al. [20], highlighted the statistically significant utility of ECA PSV in distinguishing diseased ICA conditions. These findings highlight the intricate interplay between ICA and ECA, as supported by our study, which further demonstrated these interactions both during and after CAS.

One of the key findings of our study is the occurrence of de novo ECA occlusion following CAS, observed in six patients (4.17%). Our analysis identified a greater preoperative ECA diameter as a negative predictive factor for postoperative ECA occlusion, with an OR of 0.1. Similar observations were reported by Brown et al. [21], who noted de novo ECA occlusion in 3.8% of patients following CAS. Their study also highlighted a significantly higher prevalence of de novo ECA occlusion after CAS compared to CEA (3.8% vs. 0.4%, *p* = 0.04). Interestingly, they found no significant difference in preoperative PSV between patients who developed post-CAS ECA occlusion and those who did not. These findings suggest that, while CAS is less invasive than CEA, it may still lead to complications such as de novo ECA occlusion. The absence of a significant relationship between preoperative PSV and post-CAS ECA occlusion indicates that other factors, such as the mechanical disruption caused by stent placement, could play a more substantial role in ECA occlusion. Furthermore, the higher prevalence of de novo occlusion after CAS compared to CEA could reflect the inherent differences in the procedural approach, with stent deployment potentially leading to more significant changes in ECA flow dynamics. This warrants further investigation into additional predictors of ECA occlusion, including anatomical characteristics and procedural variables.

This study shows an important findings of ECA stenosis or even de novo occlusion after CAS in the treatment of CNO. However, how are these findings correlated with clinical data? A previous study [8] found that the CAS in patients with ICA stenosis was associated with facial pain in 27.27% during the procedure and in 7.28% after 24 h from the CAS. Furthermore, the facial pain was more prevalent in patients with the lower degree of ICA stenosis. Additionally, the other clinical symptoms related to ECA stenosis are describe in the literature. In their study, Giurgea et al. [11] reported a faster time to occurrence of symptoms of jaw claudication after the procedure compared to the preprocedural time (90 s vs. 190 s). Moreover, this time increased after one week after CAS to 150 s; however, it did not return to preoperative levels. Additionally, Domanin et al. [12] reported the acute hemifacial ischemia as a long-term complication after CAS. Most importantly, Kim et al. [13] found that the presence of atherosclerotic plaque in ECA that caused the artery stenosis was an independent predictive factor for all-cause mortality, increasing the likelihood of death by 2.6 times. These findings suggest that, while CAS is effective in addressing ICA stenosis, the potential impact on the ECA should not be overlooked. The effects on the ECA could contribute to both immediate symptoms and long-term risks associated with the procedure. Therefore, a comprehensive approach considering both ICA and ECA involvement is essential to fully understand and mitigate the potential consequences of CAS.

Our study has several limitations that should be acknowledged. First, the retrospective nature of the research, combined with its single-center design, inherently limits the generalizability of the findings to broader populations and diverse clinical settings. Second, this study did not include any follow-up period, which prevents the evaluation of long-term clinical outcomes of ECA overstenting. This also precludes any detailed analysis of the progression or stabilization of ECA dynamics over time, a potentially important factor in understanding the compensatory role of the ECA after CAS. Third, our focus was exclusively on patients with CNO, explicitly excluding those with severe stenosis without near-occlusion. While this approach ensured a highly specific study population, it limits the applicability of our findings to broader populations with significant carotid artery disease. This narrow focus also excludes insights into whether CAS has differential impacts on ECA dynamics and clinical outcomes in patients with varying degrees of stenosis severity. Additionally, the inclusion and exclusion criteria, while designed to create a well-defined cohort, may have introduced selection bias. Patients with unsuccessful CAS or prior interventions were excluded, which may have resulted in the selection of cases with more favorable anatomical features or procedural outcomes. Moreover, the absence of a detailed investigation into the specific effects of ECA changes on clinical outcomes in CNO represents a significant limitation, as it leaves an important aspect of CAS in this population unexplored.

These limitations underscore the need for larger multicenter prospective studies to validate our findings. Such studies should incorporate longer follow-up periods to assess both short- and long-term outcomes, including the role of the ECA in collateral circulation and its impact on patient prognosis. Future research should also aim to include a broader spectrum of carotid artery disease severity and directly investigate the relationship between CAS-induced ECA changes and clinical outcomes to provide a more comprehensive understanding of this treatment modality.

## 5. Conclusions

This study demonstrates that CAS for CNO significantly affects the ECA, leading to a reduction in ECA diameter in most patients. The preoperative ECA diameter was found to be a key predictor of the extent of this reduction and the occurrence of postoperative ECA occlusion. Additionally, weak correlations between changes in both the ICA and ECA diameters suggest a complex relationship between these vessels during and after the procedure. A notable finding was the occurrence of de novo ECA occlusion in a subset of patients, indicating a potential complication of CAS that requires further investigation.

## Figures and Tables

**Table 1:** Group descriptive data.

Variable	*n* (%), Mean/Median (Range, SD/IQR)
Age (years)	69 (42–88) IQR 9
Gender	
Male	109 (68.55%)
Female	50 (31.45%)
Lifestyle factors	
History of Cigarette Smoking	108 (67.92%)
Current Cigarette Smoking	57 (35.85%)
Comorbidities	
Presence of comorbidities (yes)	151 (94.96%)
Generalized atherosclerosis	151 (94.96%)
Arterial hypertension	142 (89.31%)
Dyslipidemia	121 (76.10%)
Coronary artery disease	82 (51.57%)
History of myocardial infraction	64 (40.25%)
Diabetes mellitus	46 (28.93%)
Clinical symptoms	
Presence of clinical symptoms	108 (67.92%)
Stroke	66 (41.51%)
Dizziness	25 (15.72%)
Headache	14 (8.81%)
TIA	13 (8.18%)
Syncope	9 (5.66%)
Tinnitus	4 (2.51%)

Abbreviations: IQR—interquartile range, SD—standard deviation, TIA—transient ischemic attack.

**Table 2:** Perioperative patient’s data.

Variable	*n* (%), Mean/Median (Range, SD/IQR)
Preoperative assessment	
Preoperative ICA diameter (mm)	0.8 (0.2–2.7), IQR 0.36
Preoperative ECA diameter (mm)	4.34 (0.80–8.10), IQR 2.25
Preoperative ECA occlusion	7 (4.40%)
Stent characteristics	
Side of the procedure	
Left ICA	80 (50.31%)
Right ICA	79 (49.69%)
Stent used	
1st generation	68 (42.77%)
2nd generation	91 (57.23%)
Stent length	
40 mm	97 (61.01%)
30 mm	62 (28.99%)
Stent width	
6 mm	3 (1.89%)
7 mm	63 (39.62%)
8 mm	64 (40.25%)
9 mm	26 (16.35%)
10 mm	3 (1.89%)
Postoperative assessment	
Postoperative ICA diameter (mm)	5.60 (2–9.80), IQR 2
Covering of ECA orifice by a carotid stent	
Yes	144 (94.73%)
No	8 (5.26%)
ECA diameter after CAS (mm)	3.40 (0–7.70), IQR 2.00
Fate of ECA in patients with overstenting of ECA (*n* = 144)	
Decrease in the diameter	110 (76.39%)
ECA occlusion *	6 (4.17%)
No change in the diameter	24 (16.67%)
Increase in the diameter	10 (6.94%)
Difference between pre- and postoperative ECA diameter (mm)	0.9 (−2.20–4.60), IQR 1.73
Difference in post- and preoperative ICA diameter (mm)	4.92 (1.3–8.7), IQR 1.87

Abbreviations: CAS—Carotid Artery Stenting, ECA—external carotid artery, ICA—internal carotid artery, IQR—interquartile range, SD—standard deviation. Footnote: * The number of patients with ECA occlusion are also included in the decrease in the diameter number.

## Data Availability

The raw data supporting the conclusions of this article will be made available by the authors on request.
